# Schlafens: Emerging Therapeutic Targets

**DOI:** 10.3390/cancers16101805

**Published:** 2024-05-09

**Authors:** Ricardo E. Perez, Frank Eckerdt, Leonidas C. Platanias

**Affiliations:** 1 Robert H. Lurie Comprehensive Cancer Center of Northwestern University, Chicago, IL 60611, USA; ricardo.perez@northwestern.edu (R.E.P.); frank.eckerdt@northwestern.edu (F.E.); 2 Division of Hematology-Oncology, Department of Medicine, Northwestern University, Chicago, IL 60611, USA; 3 Department of Medicine, Jesse Brown Veterans Affairs Medical Center, Chicago, IL 60612, USA

**Keywords:** Schlafen (SLFN), interferon (IFN), immune therapy, cancer

## Abstract

**Simple Summary:**

The family of Schlafen (SLFN) genes are interferon-inducible genes that control various cellular processes such as anti-viral responses, RNA regulatory processes, interferon-dependent gene expression and biological responses, cell differentiation, cancer cell proliferation, and immune cell regulation. Depending on the context, some SLFNs may either mediate antitumor effects or indirectly contribute to cancer progression. Despite rapidly emerging advances in the field, the precise mechanisms by which these functionally divergent and differentially regulated SLFN family members engage in various aspects of human malignancies remain incompletely understood. Here, we highlight recent insights into the complicated roles of the SLFN family of proteins.

**Abstract:**

The interferon (IFN) family of immunomodulatory cytokines has been a focus of cancer research for over 50 years with direct and indirect implications in cancer therapy due to their properties to inhibit malignant cell proliferation and modulate immune responses. Among the transcriptional targets of the IFNs is a family of genes referred to as Schlafens. The products of these genes, Schlafen proteins, exert important roles in modulating cellular proliferation, differentiation, immune responses, viral replication, and chemosensitivity of malignant cells. Studies have demonstrated that abnormal expression of various Schlafens contributes to the pathophysiology of various cancers. Schlafens are now emerging as promising biomarkers and potentially attractive targets for drug development in cancer research. Here, we highlight research suggesting the use of Schlafens as cancer biomarkers and the rationale for the development of specific drugs targeting Schlafen proteins.

## 1. Introduction

The Schlafen (SLFN) protein family has recently attracted increasing attention for its ability to modulate cellular proliferation, differentiation, engaging immune responses, chemosensitivity, and viral and DNA replication [1,2,3,4]. Schlafen genes have been identified and described in various vertebrate species, but have mainly been studied in mice and humans. First identified in mouse thymocytes, a cluster of *Slfn* genes (*Slfn1-4*) was shown to be important for thymocyte maturation and immune development [5]. This cluster of *Slfn* genes was found expressed in lymphoid cells [5]. Since then, nine *Slfn* genes have been identified in mice (*Slfn1*, *Slfn2*, *Slfn3*, *Slfn4*, *Slfn5*, *Slfn8*, *Slfn9*, *Slfn10* pseudogene, and *Slfn14*), while in the human genome, six *SLFN* genes exist (*SLFN5*, *SLFN11*, *SLFN12*, *SLFN12L*, *SLFN13*, and *SLFN14*) [6,7]. The majority of these genes are found on chromosome 11 for mice and chromosome 17 for humans, with the exception of the Schlafen-like gene, *SLFNL1*, which is found on chromosome 4 in mice and chromosome 1 in humans. However, due to the very low similarity to other SLFNs, SLFNL1 may not be considered a ‘bona fide’ Schlafen family member [1,8,9]. Phylogenic analyses have suggested that *SLFN5* and *SLFN14* may represent orthologs shared between humans and mice (*Slfn5* and *Slfn14*, respectively) [6,9]. 

**Figure 1:**
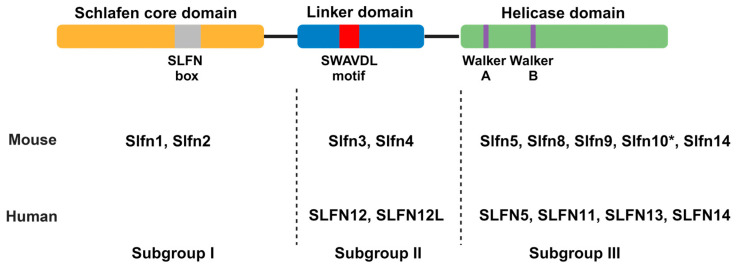
Schematic of domain compositions defining the three subgroups of Schlafen proteins. Mice and human Schlafen proteins are depicted according to their subgroup. The nuclear localization signal (NLS) found in the C-terminus of some subgroup III Schlafens is not depicted. Murine Slfn10 is a pseudogene as indicated by *. Created with BioRender.com (accessed on 30 April 2024).

Classification of SLFN proteins into three major subgroups is based on their structural and functional domains (Figure 1) [7,10,11]. As the nomenclature of SLFN domains has shifted over the years, we have decided to adopt the domain classification as inferred from the most recent cryoEM structure, i.e., Schlafen core domain, linker domain, and helicase domain [12,13]. Subgroup I consists of SLFN proteins that exhibit only the Schlafen core domain, consisting of a nuclease domain and the unique SLFN sequence referred to as the “SLFN box”, conserved in all SLFNs. Currently, there is no known biological function for the SLFN box, but this may change in the future. Subgroup II SLFNs additionally exhibit a linker domain with the SWAVDL motif, also of unknown function. Only subgroup III SLFNs additionally exhibit a C-terminal helicase domain with striking similarity to superfamily I (SF I) DNA/RNA helicases, including Walker A/B motifs [10]. Additionally, a nuclear localization signal (NLS) can be found at the C-terminus of long SLFNs leading to their predominate localization in the nucleus with the exception of human SLFN13 and SLFN14 [3,7,14,15]. SLFNs of subgroups I and II have been found to localize to the cytoplasm in both mice and humans. Mice express all three subgroups compared to humans that only express SLFNs from subgroups II and III. 

Additionally, a virus-specific SLFN ortholog has been identified v-Slfn, resembling the Schlafen-like protein SLFNL1, found in mice and humans, containing the SLFN box and a partial Schlafen core domain [16]. 

## 2. Schlafen 5

Based on the Cancer Genome Atlas (TCGA), genes belonging to the Schlafen family are aberrantly expressed in the majority of cancers [17]. This has sparked interest in studying SLFNs to better understand and define their role in malignant transformation and their potential as targets for the treatment of different cancers. Our laboratory has previously shown that interferons (IFNs), specifically type I IFNs, can induce the expression of SLFNs in various cancers, including glioblastoma (GBM), pancreatic cancer, and melanoma [18,19,20]. Of the six *SLFN* genes, Schlafen 5 (*SLFN5*) has been consistently shown to be induced by type I IFNs in these cancers [18,19,20], as well as in triple-negative breast cancer, as shown by others [18,19,20,21]. In GBM, SLFN5 impedes STAT1-mediated transcription of interferon-stimulated genes (ISGs) following type I IFN treatment, creating a negative feedback loop as *SLFN5* is both itself an ISG and a negative regulator of IFN responses [18]. Additionally, deletion of SLFN5 triggers enhanced expression of ISGs, resulting in greater antitumor activity, as observed in GBM patient-derived cell lines grown as neurospheres under stem cell permissive conditions. On the other hand, in malignant melanoma cells, SLFN5 appears to play a different role, suppressing anchorage-independent growth and invasion of cancerous melanoma cells [20]. In that context, our group has previously demonstrated the importance of SLFN5 expression for the antitumor effects of IFNα in malignant melanoma cells [20]. In these cells, IFN-α2 treatment induced expression of *SLFN5* but no other *SLFNs*, suggesting a distinct role for SLFN5 in IFN responses in melanoma. These observations demonstrate a cancer-specific function of SLFN5 in tumorigenesis and IFN responses. 

Beyond type I IFNs, other mechanisms have been identified that result in the upregulation of certain SLFN genes in various cancers. For example, in castration-resistant prostate cancer (CRPC), *SLFN5* was identified to be highly upregulated [22]. The high amounts of *SLFN5* were found associated with poor outcomes in CRPC patients. Accordingly, SLFN5 regulates the expression of the amino acid transporter LAT1 through SLFN5’s association with the transcription factor ATF4. The loss of SLFN5 leads to lower intracellular amounts of essential amino acids and decreased mTORC1 signaling in a LAT1-dependent manner. Additionally, the loss of human SLFN5 leads to decreased tumor growth in vivo. These observations suggest that SLFN5 may be a possible target for the treatment of CRPC. Recently, Wang et al. established that the expression of SLFN5 was increased in the human lung cell line A549 and a mouse lung pneumonia model by the glycolipid lipopolysaccharide (LPS), an endotoxin produced by Gram-negative bacteria, and the cause for the inflammatory response seen in lungs affected by pneumonia [23]. Knockdown-mediated decreased expression of SLFN5 resulted in lower expression of the inflammatory markers TNF-α, IL-1β, and IL-6 [23]. These similar observations from an LPS-induced pneumonia mouse model and human A549 cells provide partial evidence that the human and mouse *SLFN5* gene products are functional orthologs, at least in the context of LPS-mediated pulmonary inflammation. 

Work from our group has provided evidence that SLFN5 is important for the tumorigenesis of pancreatic ductal adenocarcinoma (PDAC), an aggressive cancer with poor outcomes. In PDAC, high *SLFN5* levels correlate with poor survival [19]. CRISPR knockout of *SLFN5* in pancreatic cancer cells results in decreased PDAC growth in vitro and in vivo, supporting the hypothesis that SLFN5 is required for PDAC growth [19]. In that study, we also demonstrated that the association between SLFN5 and the transcriptional regulator E2F7, a known transcriptional repressor of genes required for proper S phase progression, blocked E2F7’s ability to bind to the promoters of *E2F1* and *CDC6* [19]. Additional evidence supports the idea that SLFN5 is linked with other transcription factors to provide proliferation and oncogenic cellular programs in PDAC. For instance, Weismueller et al. demonstrated that ZNF154 promoter methylation status correlated with postoperative survival [24]. Patients whose malignant cells had methylation at the ZNF154 promoter had a favorable postoperative survival compared to those promoters that were unmethylated. Furthermore, exogenous expression of ZNF154 in pancreatic cancer cells revealed increased SLFN5 expression, indicating a novel link between SLFN5 and ZNF154. Altogether, Weismueller et al. [19] and Fischietti et al. [25] reinforced the importance of SLFN5 for pancreatic cancer development and the potential for targeting SLFN5 in pancreatic cancer [19,24]. Further, in ovarian cancer, a highly malignant gynecological cancer, SLFN5 was linked to the ability of ovarian cancer cells to migrate and invade [25]. In these cells, SLFN5 regulates the epithelial to mesenchymal transition (EMT), specifically reducing expression of the transmembrane glycoprotein E-cadherin, leading to increased migration and invasion [25]. Inversely, SLFN5 has been associated with inhibition of motility and invasion of renal cell carcinoma cells. Specifically, SLFN5 inhibits the expression of known cell motility genes *MMP-1* and *MMP-13* [26]. Moreover, the expression of MT1-MMP was repressed by over-expressing SLFN5 in the fibrosarcoma cell line HT1080 and the renal clear cell cancer cell line 786-0 diminishing the ability of these cells to migrate and invade [27]. In summary, SLFN5 can both promote or inhibit the ability of cancer cells to migrate and invade in a cell/tissue-dependent manner (Table 1). These contrasting regulatory functions need to be further explored to better understand SLFN5’s potential as a therapeutic target.

The context-dependent and sometimes conflicting, observations reported for SLFN5 regulatory effects, are also evident in different types of breast cancer. SLFN5 is expressed in estrogen receptor (ER)-positive breast cancer cells, while triple-negative breast cancer (TNBC) cells lack SLFN5 or exhibit very low levels [29,30]. Over-expressing SLFN5 in TNBC cells resulted in the re-expression of the tumor suppressor PTEN leading to a decrease in the AKT/GSK3β/β-catenin pathway and TNBC aggressiveness [29], whereas in MCF7, a low invasive breast cancer cell line, knockdown of SLFN5 increased activation of the AKT/GSK3β/β-catenin pathway that leads to increased expression of MT1-MMP and enhanced migration and invasion [27]. The transcription factor ZEB1, a known regulator of EMT, was shown to repress *PTEN* expression in breast cancer cells. Meanwhile, SLFN5 expression inhibits ZEB1 repression of *PTEN* by binding to the *ZEB1* promoter and inhibiting its transcription [29]. Similar to ovarian cancer, where SLFN5 stimulates EMT (and migration and invasion) through repression of E-cadherin [25], SLFN5 also promotes migration and invasion in MCF7 cells by transcriptional repression of *ZEB1* [30], indicating a conserved cancer-promoting role for SLFN5 by stimulating EMT in epithelial cancers. However, work from the Lu group on the role of SLFN5 in breast cancer cells shows that SLFN5 could be considered a tumor suppressor in the context of TNBC by inhibiting the aggressiveness commonly seen by TNBC [27,29,30]. Further studies are necessary to better elucidate the opposing roles of SLFN5 in TNBC versus other types of breast cancer. 

## 3. Schlafen 11

There is evidence that Schlafen 11 (SLFN11) can be induced by type I IFNs in non-transformed cells and certain immune cells [31,32]. However, several lines of evidence suggest that SLFN11 is also induced by IFNγ, a type II IFN [18,20,33]. The role of SLFN11 in IFNγ mediated toxicity seems to be context-dependent because SLFN11 was shown to be required for IFNγ mediated toxicity in HAP1 cells but not in prostate or melanoma cell lines [33]. In HAP1 cells, SLFN11 did not suppress the induction of other IFNγ inducible genes [33]. Rather, SLFN11 may stimulate the induction of immune-related gene expression (including genes of the IFNγ signaling pathway) in response to DNA-damaging agents (DDAs) [34]. Thus, SLFN11 does not seem to be part of a conserved negative feedback regulatory loop because it does not generally repress type II IFN-mediated transcriptional responses. 

SLFN11 represents another member of subgroup III of SLFNs and has widely been considered a predictive biomarker for cancer cell chemosensitivity to various DDAs. A lack of SLFN11 expression renders a variety of cancers resistant to certain DDAs, as was demonstrated for topoisomerase I and II inhibitors, alkylating agents, and DNA synthesis inhibitors (e.g., gemcitabine) [35]. The sensitivity to these agents was linked to SLFN11 recruitment to areas of damaged DNA by the replication protein A1 (RPA1) resulting in destabilization of RPA1-single-stranded DNA (ssDNA), leading to cell death due to impaired homologous repair (HR) [36]. In addition to these DDAs, elevated expression of SLFN11 also correlated with sensitivity to poly-(ADP)-ribose polymerase inhibitors (PARPi) [35]. PARPs mediate DNA damage responses and small cell lung cancer (SCLC) PDXs lacking SLFN11 expression were found to be resistant to the PARPi talazoparib as compared to PDXs that expressed high levels of SLFN11. Another pathway responding to replication stress is the DNA sensor ataxia telangiectasia and Rad3-related protein (ATR) pathway [37]. ATR transiently arrests cells through its target Chk1 by inducing an intra-S-phase arrest until the replication stress is resolved [37,38]. The Pommier group has demonstrated that SLFN11 could inhibit stressed replication forks independent of ATR and this blockage-induced cancer cell death by SLFN11 was deemed irreversible [4,39]. Work from several groups has demonstrated the reliance on the ATR/Chk1 pathway in cancers that express little to no SLFN11, making these cancers more susceptible to ATR inhibition [39,40,41]. Therefore, SLFN11 may serve as a diagnostic marker, as cancers with low SLFN11 expression are expected to be highly vulnerable to pharmacological inhibition of ATR pathway components when exposed to DDAs.

Our group demonstrated in GBM that SLFN11 suppresses noncanonical NFκB signaling allowing for GBM progression [42]. We also demonstrated an association between SLFN11 and NFκB2, resulting in repression of the cell cycle blocker p21 [42]. Loss of SLFN11 stimulated NFκB2 dependent expression of p21, blocking GBM growth as demonstrated by increased survival of orthotopic PDXs lacking SLFN11. Additionally, GBM patients who have high SLFN11 levels exhibit worse overall survival [42]. Recently, SLFN11 has been linked to the malignant phenotype of clear cell renal cell carcinoma (ccRCC) promoting tumor growth, migration, and invasion [43]. *SLFN11* is highly overexpressed in ccRCC where it is associated with poor overall survival [44]. Wang et al. demonstrated that SLFN11 promotes the PI3K/AKT pathway supporting the increase of cellular proliferation, migration, and invasion as compared to ccRCC cells that lack SLFN11. The antineoplastic effects after the loss of SLFN11 and its effects on PI3K/AKT signaling were rescued with 740 Y-P, a PI3K agonist [43]. The authors of this work have suggested that SLFN11 could be considered an oncogene in ccRCC. The evolution of SLFN11 as an oncogene needs to be further investigated. By contrast, in hepatocellular carcinoma (HCC) patients, decreased expression of *SLFN11* was linked to worse overall survival and increased recurrence [45]. Zhou et al. over-expressed SLFN11 in HCC cell lines and found an interaction between SLFN11 and the oncogenic ribosomal protein S4 X-linked (RPS4X) [45]. RPS4X levels were shown to be inversely correlated with SLFN11 in HCC tumor tissue. The interaction between SLFN11 and RPS4X inhibits the mTOR signaling pathway as judged by decreased phosphorylation of S6 and eIF4E. Pharmacologic mTOR inhibition or over-expressing SLFN11 induced apoptosis of HCC cell lines [45].

## 4. Schlafen 12

Schlafen 12 (SLFN12) is a member of subgroup II of SLFNs that tends to localize primarily in the cytoplasm [7]. SLFN12 lacks the helicase domain and NLS present in the C-terminus in most subgroup III SLFNs [6]. The rodent Slfn3 and human SLFN12 exhibit overlapping roles in small intestinal epithelial differentiation [46,47]. Similar to SLFN5, SLFN12 can regulate the production of the transcription factor ZEB1 in TNBC, lessening the aggressiveness associated with TNBC. SLFN12 reduces protein levels of ZEB1 by attenuating ZEB1 translation and inducing its proteasomal degradation [48]. RNA-seq analysis of SLFN12-over-expressing xenografts reveals expression of less aggressive breast cancer markers HER2 receptors ERBB2 and EGFR that usually are absent in TNBC. Additionally, SLFN12-over-expressing xenografts express markers of a differentiated and thus less aggressive luminal phenotype [49]. Similarly, SLFN12 impedes c-myc translation in lung adenocarcinoma (LUAD) but not lung squamous cell carcinoma (LSCC), resulting in reduced proliferation of LUAD [50]. In both TNBC and LUAD, increasing the expression of SLFN12 may contribute to better therapeutic options.

The majority of compounds that modulate SLFN12 are a class of small molecules referred to as velcrins. These molecules induce a heterotetramer complex between SLFN12 and the protein phosphodiesterase 3A (PDE3A) [51,52]. The complex induces cell killing by stabilizing the SLFN12 protein, which requires its N-terminal RNase activity. Recently, the Greulich group has demonstrated that the velcrin-induced PDE3A-SLFN12 complex digests the tRNA^Leu^(TAA), inducing impairment in global translation leading to apoptosis [53]. Along with velcrins, 17-β-estradiol (E2) and its related hormones (e.g., testosterone, progesterone, corticosterone) bind to PDE3A and can stabilize SLFN12 to form the PDE3A-SLFN12 complex promoting cell death through repression of the anti-apoptotic proteins Bcl-2 and Mcl-1. The PDE3A-SLFN12 complex formed by E2 binds to ribosomes at the endoplasmic reticulum and prevents protein translation [54]. Until now, the targeting of SLFN12 through the use of velcrins has shown great potential for the treatment of SLFN12- and PDE3A-positive cancers. 

The SLFN family of proteins has been greatly investigated as IFN inducible genes. SLFN5 seems to be primarily induced in the majority of cells by type I IFNs while the transcriptional induction of other SLFN proteins by IFNs seems to be cell-type-dependent. For instance, type I IFNs induced the expression of SLFN5, SLFN11, SLFN12, and SLFN13 in normal melanocytes, but in melanoma cell lines, only SLFN5 was inducible [20]. Similarly, in various GBM cell sources, only *SLFN5* was consistently induced by type I IFN treatment, while the inducible expression of other SLFNs was more variable [18]. In efforts to investigate the intra-play between the SLFN proteins, Brown et al. recently suggested SLFN12 may promote the IFN-induced expression of SLFN11 and SLFN13 in MDA-MB-231 TNBC cells, while not affecting the induction of SLFN5, SLFN12L, and SLFN14 in response to IFN-α2 treatment [21]. However, these results were not fully recapitulated in additional TNBC cell lines, corroborating the notion of context-dependent variability in transcriptional regulation of distinct SLFN family members [21]. Still, loss of SLFN12 did not inhibit the loss of cellular viability by IFN-α2 treatment suggesting that IFN-α2-induced cellular death is not dependent on SLFN12. There is evidence that SLFN12 may be needed for SLFN11 re-expression for greater sensitivity to certain chemotherapy agents [21], and overexpression data may indicate that SLFN12 itself may serve as a predictive biomarker for sensitivity to chemotherapy [55]. Moreover, mouse Slfn3 has also been linked to the expression of other *Slfn* family members in the ileum, thymus, and spleen of mice [56]. These studies suggest that SLFN12/Slfn3 may be required for controlled expression of other SLFN/Slfn in response to certain stimuli. 

## 5. Schlafens and Immune Cells

Schlafens were first discovered in mouse immune cells, and they were shown to regulate T cell differentiation and maturation. Specifically, Slfn1 maintains T cell quiescence through inducing cell cycle arrest [5]. A non-functional Slfn2 (Elektra mouse) results in immunodeficient mice through the low number of CD4+ and CD8+ T cells [57]. In human immune cells, SLFN levels were registered in primary T cells, monocytes, and monocytic-derived dendritic cells (moDCs) [32]. *SLFN5*, *SLFN12L*, and *SLFN13* levels were highly expressed in primary T cells. Meanwhile, *SLFN11* levels were primarily expressed in monocytes and moDCs [32]. Upon differentiation of monocytes to moDCs, *SLFN12L* and *SLFN13* levels were upregulated while *SLFN12* levels were lowered in moDCs upon differentiation from monocytes. Activation of T cells by CD3 or CD3/CD28 resulted in increased *SLFN5*, *SLFN12*, *SLFN12L*, and *SLFN13* following induction by IFNα [32]. *SLFN11* was not induced following treatment with IFNα in activated T cells and this is similar to observations from cancer cells where IFNα induced expression of most SLFNs but not SLFN11, suggesting an altered activation pathway for *SLFN11*. 

Previously, Ding et al. demonstrated that SLFN4+ myeloid-derived suppressor cells (MDSCs) could migrate to the stomach following *Helicobacter pylori* infection potentiating the development to gastric metaplasia [58]. Similarly, SLFN12L (which exhibits sequence similarity with Slfn4) has been shown to colocalize with MiR130b, a microRNA that was associated with SLFN4+ MDSCs that suppressed T cells and promoted *Helicobacter*-induced metaplasia [59]. The levels of SLFNs in gastric cancer were screened from the TCGA database and all *SLFNs* except *SLFN14* were upregulated compared to normal gastric tissue but only *SLFN5* and *SLFN13* were found to associate with poor overall survival [60]. 

In high-grade serous ovarian cancer (HGSOC), SLFN11 expression was found to be highly present in macrophages and monocytes, while in neutrophils, SLFN11 expression was barely detected. Increased expression of SLFN11 in noncancerous (tumor-infiltrating lymphocytes, TILs) was strongly associated with better overall outcomes. Upon activation, T cells in HGSOC increase expression of SLFN11, as is similarly seen following antigen presentation [34]. Winkler et al. suggested that SLFN11 could be considered a dual biomarker for the sensitivity of HGSOC to platinum agents and the immunological infiltration and activation [34]. Similarly, in RCC, SLFN11 was strongly associated with TILs such as T cells, macrophages, and dendritic cells. Additionally, Liu et al. correlated the increased expression of immune checkpoint genes CTLA4 and CD244 with SLFN11. These associations indicate immune regulatory roles for SLFN11 that may have clinical translational implications for cancers suffering from immunosuppressive microenvironment [44].

## 6. Conclusions

Recently, the family of Schlafen proteins has attracted attention in the cancer field, implicated in various aspects of cancer biology. Despite remarkable advancements in cancer treatment with immune checkpoint inhibitors (ICIs), there are several cancers that are considered immunologically “cold” or do not respond well to immunotherapy, with a variety of mechanisms accounting for that lack of response [61,62]. We have previously proposed that SLFN5 may act as a potential “intracellular immune checkpoint” by inhibiting IFN-signaling [63]. Targeting SLFN5 in certain cancers could potentially help optimize the tumor microenvironment, facilitating the recruitment of anti-tumor immune cells. Further understanding of the function of human SLFNs in immune cells may facilitate the development of strategies that stimulate the recruitment of these cells to the tumor microenvironment. Developing approaches to target SLFN5, and potentially other SLFNs, may result in the emergence of a unique way to overcome immunotherapy resistance. On the other hand, it is also possible that some malignancies could benefit from upregulating the expression of certain SLFN family members. For instance, the re-expression of SLFN11 in cancer cells that have silenced its expression through various mechanisms of transcriptional silencing could increase sensitivity to a variety of DDAs [64,65,66]. The complexity of the system and the potentially substantial impact in developing new therapeutic approaches underscores the importance of studies to precisely define the function of individual human SLFNs in immune activation and tumor biology. Moreover, further characterization of SLFNs as biomarkers for cancer treatment may also have important clinical implications. For instance, there is emerging evidence for the relevance of SLFN11 expression as a biomarker for chemosensitivity and homologous recombination repair [67,68]. Further studies may uncover important additional biomarker roles for these proteins in the treatment of malignancies and other human disorders.

## Figures and Tables

**Table 1:** Divergent functional roles of SLFN5 in different tumor types.

Cancer Type	Proposed Mechanism	References
Gastric	Promotes intestinal metaplasia to cancer	[28]
Breast	Inhibition of AKT signaling-Antitumor effects.	[29,30]
Renal Cell Carcinoma	Suppression of motility and invasion	[26]
Melanoma	Inhibition of invasion	[20]
Glioblastoma	Promotion of proliferation and invasion	[18]
Pancreatic Cancer	Promotion of proliferation and regulatory effects on cell cycle progression	[19]
Prostate	Promotion of cell migration through increased LAT1 expression	[22]
Ovarian	Promotion of epithelial-mesenchymal transition	[25]

## Data Availability

No new data were created or analyzed in this study. Data sharing is not applicable to this article.
